# Endovascular stenting to treat an acute stroke caused by severe stenosis of the duplicated middle cerebral artery

**DOI:** 10.1186/s12883-023-03456-4

**Published:** 2023-11-20

**Authors:** Lei Wang, Zhiyong Zhang

**Affiliations:** Department of Neurology, Beijing Geriatric Hospital, Beijing, China

**Keywords:** Duplicated middle cerebral artery, Endovascular stenting, Stenosis, Acute ischemic stroke, Progressive stroke

## Abstract

**Background:**

The duplicated middle cerebral artery (DMCA), a rare anatomical variant of the middle cerebral artery, arises between the anterior choroidal artery and the distal end of the internal carotid artery. We present the case of a patient who had an acute progressive stroke caused by severe stenosis in the initial segment of the DMCA and was successfully treated with endovascular stenting.

**Case presentation:**

A 57-year-old man was admitted to our hospital with sudden left extremity weakness for three days. Cranial magnetic resonance imaging revealed multiple fresh infarcts in the right basal ganglia and temporal lobe. Cerebrovascular imaging revealed severe stenosis of the right DMCA’s initial segment. However, despite standard medical therapy, the patient’s limb weakness worsened. Based on the clinical and imaging findings, we speculated that severely stenotic DMCA is responsible for the acute progressive stroke. On the basis of the best medical treatment, the patient successfully underwent endovascular stent implantation under general anesthesia two weeks after the onset. The patient’s condition was stable after interventional therapy, and his postoperative follow-up prognosis was favorable.

**Conclusions:**

Endovascular stenting may be a feasible treatment for symptomatic severe stenosis of the DMCA in cases of poor control with standard medications.

## Background

A duplicated middle cerebral artery (DMCA), a rare vascular anomaly of the middle cerebral artery (MCA) [1], was first described by Crompton in 1962 [2]. For many years, DMCA was considered to have no clear clinical significance [3]. However, recent cases have demonstrated a close relationship between DMCA detection and aneurysm development [4]. In contrast, the association between DMCA and ischemic cerebral disease has rarely been reported [5], and only a few reports have focused on the collateral circulation role of DMCA in the event of main MCA occlusion. Previous studies have reported that medical therapy is predominantly used to treat DMCA stenosis [3, 4]. To the best of our knowledge, endovascular therapy for managing such stenoses has not yet been reported. We describe a patient with symptomatic severe DMCA stenosis who was successfully treated with endovascular stenting and also discuss the literature relevant to this case.

## Case presentation

**Fig. 1:**
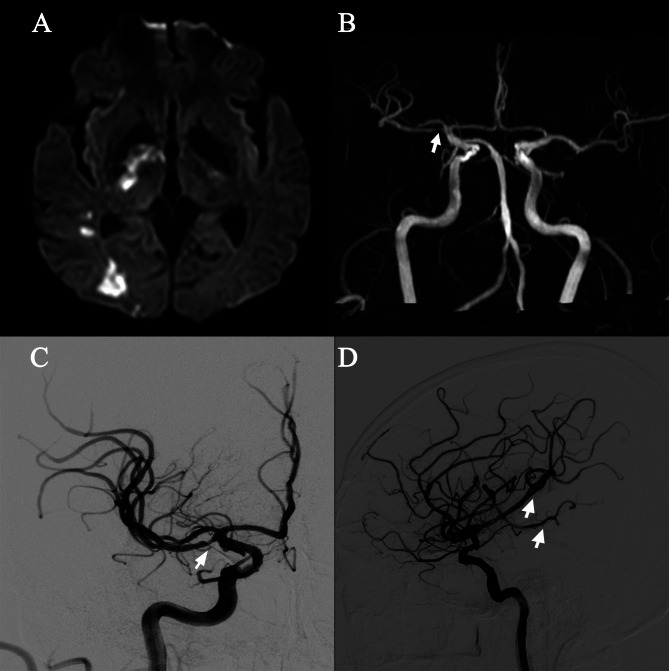
**(A)** DWI-sequence axial MRI showed multiple fresh infarcts in the right basal ganglia and temporal lobe. **(B, C)** MRA and DSA revealed that the DMCA (arrow) originated from the distal end of the ICA, with stenosis at the origin. **(D)** DMCA’s branches in the lateral view of the angiography (arrow)

A 57-year-old right-handed man was admitted to the Department of Neurology in our hospital due to sudden-onset left-limb weakness that began three days prior. He denied other concomitant symptoms, such as dizziness, headache, diplopia, aphasia, limb tics, and impaired consciousness. The patient’s medical history included poorly controlled hypertension and long-standing type 2 diabetes. Personal and familial histories were unremarkable. Neurological examination after admission showed a left-limb muscle strength of 4/5 with a positive Babinski sign (National Institute of Health Stroke Scale, NHISS = 2). Laboratory parameters were within the normal ranges, except for hyperlipemia and hyperhomocysteinemia, and there were no cardiac abnormalities on both the physical examination and on routine electrocardiography, Holter electrocardiography, transthoracic echocardiography, and transesophageal echocardiography. Cranial magnetic resonance imaging (MRI) showed multiple fresh infarcts in the right basal ganglia and temporal lobe (Fig. 1A). Magnetic resonance angiography (Fig. 1B) and digital subtraction angiography (DSA) (Fig. 1C) revealed a right DMCA originating from the distal end of the internal carotid artery (ICA). Its course and diameter were parallel to and equal to those of the main MCA, respectively. In addition, there was severe stenosis at the initial segment of the DMCA. Figure 1D showed the branches of the DMCA. Other intracranial and extracranial vessels showed no obvious abnormalities.

**Fig. 2:**
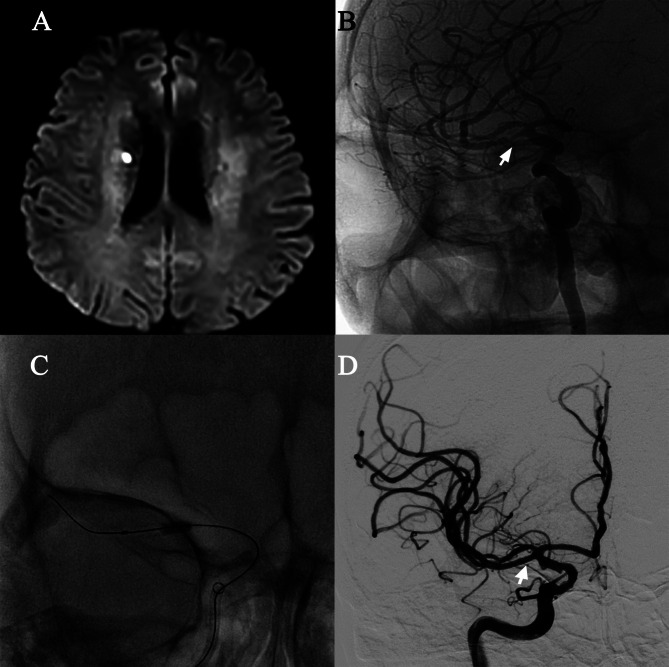
**(A)** DWI-sequence axial MRI showed a new infarct in the right corona radiata. **(B)** 3D-DSA imaging. **(C)** The stent was successfully released at six atmospheric pressures. **(D)** The stenosis disappeared after the DMCA stent implantation (a 2.25/10 mm NOVA intracranial sirolimus-eluting stent system, SINOMED, Tianjin, China)

After admission, the patient was treated with 100 mg of aspirin, 75 mg of clopidogrel, and 40 mg of atorvastatin once daily, and other risk factors for cerebrovascular disease were controlled. Unfortunately, his condition worsened three days after admission, and his left-limb muscle strength decreased to 3/5 (NHISS = 4). A repeat cranial MRI revealed a new infarct in the right corona radiata (Fig. 2A). Based on the above findings, we believed that the right DMCA was the vessel responsible for the acute stroke. In addition to pharmacotherapy, DMCA stent implantation was performed under general anesthesia 14 days after onset.

The diameter of the normal vessel at both ends of the stenosis, the diameter of the stenosis, and the length of the stenosis were precisely measured by 3D-DSA (Fig. 2B) and were found to be 2.3 mm, 0.6 and 8 mm, respectively. Under general anesthesia, 3000 units of heparin were intravenously administered to the patient. A 6 F TracLine™ guiding catheter (HEMO, Weihai, Shandong, China) was introduced through an 8 F femoral sheath into the C1 segment of the right ICA. Then a 6 F 125-cm intracranial support catheter (TONBRIDGE MEDICAL, Zhuhai, Guangzhou, China) was introduced into the C3 segment of the right ICA. Under an optimal road map for the severely stenotic right DMCA, a 2.25/10 mm NOVA intracranial sirolimus-eluting stent (SINOMED, Tianjin, China) guided by a Synchro^2^ microguidewire (Stryker, Salt Lake City, Utah, USA) via the support catheter was placed in the right DMCA’s stenotic segment. Finally, the stent was successfully released at six atmospheric pressures after the precise positioning by the super-selective angiography (Fig. 2C and D).

Fortunately, no perioperative complications occurred, such as embolism, arterial spasm, acute arterial occlusion, hyperperfusion syndrome, arterial dissection, and cerebral hemorrhage. Following the placement of the drug-eluting stent, this patient received oral aspirin 100 mg daily and clopidogrel 75 mg daily for 90 days before converting to aspirin 100 mg daily [6]. After three weeks of hospitalization, his left-limb muscle strength increased to 5-/5 (NHISS = 0). Follow-up for three months showed a good prognosis without a recurrent stroke event (modified Rankin Scale, mRS = 0).

## Discussion and conclusions

MCA anomalies occur less frequently than anomalies of the other major intracranial arteries. MCA anomalies consist of accessory MCA, DMCA, duplicated origin of MCA, fenestration of MCA, and unfused or twig-like MCA [7]. The incidence of accessory MCA is 0.3-4.0%, whereas the incidence of DMCA is lower at 0.2–2.9% [2, 5, 8]. In 2011, Chang et al. divided DMCAs into two types: type A (DMCAs originating from the top of the ICA) and type B (DMCAs arising between the top of the ICA and the anterior choroidal artery). The diameter of type A-DMCA is the same or slightly shorter than that of the main MCA, whereas the diameter of type B-DMCA is the same, slightly smaller, or much smaller than that of the main MCA. Type A-DMCA shows a course parallel to that of the main MCA, whereas type B-DMCA shows a parallel course or curves toward the temporal lobe [3]. In the present case, angiography revealed that the DMCA originated at the top of the ICA. In addition, it had a parallel course and an equal diameter to those of the main MCA, indicating that it was a type A-DMCA.

DMCAs are rare congenital variations of the MCA and are considered to have no clinical significance [3]. Along with the increasing number of case reports of aneurysms at the origin of the DMCA, an association between DMCAs and cerebral aneurysms has been well documented [9]. However, the possible pathogenesis remains unclear. Shear stress from blood pressure [10], elastin discontinuity with a thinned sub-endothelium, and disruption of the internal elastic lamina and media may contribute to the aneurysm [3, 11]. Similar to the main MCA, the DMCA supplies the basal ganglia and temporal lobe [5, 7, 12]. However, DMCA-related cerebral infarctions are rare [5]. A previous study that explored the etiology of accessory/DMCA-related cerebral infarction found that strokes are primarily caused by embolism (including cardiogenic embolism and embolic stroke of undetermined source) followed by cerebral artery dissection and atherothrombotic infarction. A thorough cardiac examination ruled out cardiogenic embolism in this case. On cranial magnetic resonance imaging, new infarct lesions were scattered in the right temporal lobe and basal ganglia, and there was no definite stenosis in the right anterior circulation system, except for severe DMCA stenosis. Therefore, the culprit vessel was considered to be the DMCA. According to the Trial of ORG 10,172 in Acute Stroke Treatment (TOAST) criteria, the cause of this stroke is first considered to be large-artery atherosclerosis due to the presence of multiple cerebrovascular disease risk factors, and stenotic DMCA without imaging changes like arterial dissection and vasculitis.

Current guidelines for symptomatic severe intracranial atherosclerotic stenosis recommend optimal medical treatment, especially dual antiplatelet and intensive statin therapy, and consider endovascular treatment as an option for failed medical therapy [13]. This patient’s symptoms continued to progress despite standard medical treatment, meeting the guideline recommendations for endovascular treatment. Unlike other large intracranial vessels, the DMCA may have a slender diameter, an unusual anatomical orientation, and a possibly defective vessel wall structure [5, 14]; therefore, endovascular treatment needs to be more fully evaluated. In this case, progressive stroke was caused by severe DMCA stenosis, and the normal diameter on both sides of the stenosis was relatively large. The stenotic vascular segment was straight, and the patient had no intracranial aneurysms associated with the DMCA. Thus, there was a definitive indication for endovascular therapy. A randomized clinical trial conducted in 2022 revealed that drug-eluting stents were superior to standard bare-metal stents in reducing in-stent restenosis and stroke recurrence in patients with symptomatic high-grade intracranial atherosclerotic stenosis. This led to a preference for the NOVA intracranial sirolimus-eluting stent system [6]. Endovascular stent implantation under general anesthesia was successfully performed after a comprehensive evaluation.

To our knowledge, this is the first case report in which severe DMCA stenosis was successfully treated by endovascular stenting. Our study may have a potential revelatory effect on the interventional diagnosis and treatment of DMCA-related ischemic disease, and we expect that more relevant cases or studies will be published in the future.

## Data Availability

The corresponding author can be directly contacted to apply for permissions to obtain access to the raw data.

## Abbreviations

DMCA: Duplicated middle cerebral artery
MCA: Middle cerebral artery
NHISS: National Institute of Health Stroke Scale
MRI: Magnetic resonance imaging
ICA: Internal carotid artery
mRS: Modified Rankin Scale
TOAST: Trial of ORG 10172 in Acute Stroke Treatment

## References

[1] Otsuka N, Yajima H, Miyawaki S, Koizumi S, Kiyofuji S, Hongo H, Teranishi Y, Kin T, Saito N (2022). Case Report: clipping an internal carotid artery Aneurysm with a duplicated Middle cerebral artery and the Anterior Choroidal Artery arising from the Dome. Front Neurol.

[2] Crompton MR (1962). The pathology of ruptured middle-cerebral aneurysms with special reference to the differences between the sexes. Lancet (London England).

[3] Chang HY, Kim MS (2011). Middle cerebral artery duplication: classification and clinical implications. J Korean Neurosurg Soc.

[4] Gao F, Jiang WJ (2009). Transient ischemic Attack associated with stenosis of accessory middle cerebral artery: a case report. Clin Neurol Neurosurg.

[5] Tsuyama K, Miyamoto N, Shindo A, Hira K, Ueno Y, Yatomi K, Oishi H, Hattori N. Analysis for Stroke etiology in Duplicated/Accessory MCA-Related cerebral infarction: two case report and brief literature review. Diagnostics (Basel Switzerland) 2021, 11(2). 10.3390/diagnostics11020205 PMC7912107 33573270

[6] Jia B, Zhang X, Ma N, Mo D, Gao F, Sun X, Song L, Liu L, Deng Y, Xu X (2022). Comparison of drug-eluting stent with Bare-Metal Stent in patients with symptomatic high-grade intracranial atherosclerotic stenosis: a Randomized Clinical Trial. JAMA Neurol.

[7] Uchiyama N (2017). Anomalies of the Middle cerebral artery. Neurologia medico-chirurgica.

[8] Umansky F, Dujovny M, Ausman JI, Diaz FG, Mirchandani HG (1988). Anomalies and variations of the middle cerebral artery: a microanatomical study. Neurosurgery.

[9] Imahori T, Mizobe T, Fujinaka T, Miura S, Sugihara M, Aihara H, Kohmura E (2020). An Aneurysm at the origin of a duplicated Middle cerebral artery treated by stent-assisted coiling using the wrapped-Candy Low-Profile visualized Intraluminal support (LVIS) technique: a technical Case Report and Review of the literature. World Neurosurg.

[10] Debette S, Compter A, Labeyrie MA, Uyttenboogaart M, Metso TM, Majersik JJ, Goeggel-Simonetti B, Engelter ST, Pezzini A, Bijlenga P (2015). Epidemiology, pathophysiology, diagnosis, and management of intracranial artery dissection. Lancet Neurol.

[11] Finlay HM, Canham PB (1994). The layered fabric of cerebral artery fenestrations. Stroke.

[12] Komiyama M, Nakajima H, Nishikawa M, Yasui T (1998). Middle cerebral artery variations: duplicated and accessory arteries. AJNR Am J Neuroradiol.

[13] Gutierrez J, Turan TN, Hoh BL, Chimowitz MI (2022). Intracranial atherosclerotic stenosis: risk factors, diagnosis, and treatment. Lancet Neurol.

[14] Turk AS, Ahmed A, Niemann DB, Aagaard-Kienitz B, Brooks N, Levine RL (2007). Utilization of self-expanding stents in the treatment of intracranial atherosclerotic Disease in the distal small cerebral vessels. Neuroradiology.

